# Rice nitrogen nutrition monitoring classification method based on the convolution neural network model: Direct detection of rice nitrogen nutritional status

**DOI:** 10.1371/journal.pone.0273360

**Published:** 2022-11-22

**Authors:** Qiang Zhai, Chun Ye, Shuang Li, Jizhong Liu, Zhiming Guo, Ruzhi Chang, Jing Hua

**Affiliations:** 1 School of Mechatronic Engineering, Nanchang University, Nanchang City, Jiangxi Province, China; 2 Institute of Agricultural Engineering/Jiangxi Province Engineering Research Center of Intelligent Agricultural Machinery Equipment/Jiangxi Province Engineering Research Center of Information Technology in Agriculture, Jiangxi Academy of Agricultural Sciences, Nanchang, China; 3 Weichai Power Co., Ltd., WeiFang City, ShanDong Province, China; 4 School of Software, Jiangxi Agricultural University, Nanchang City, Jiangxi Province, China; National University of Computer and Emerging Sciences, PAKISTAN

## Abstract

The nitrogen nutrition status affects the main factors of rice yield. In traditional rice nitrogen nutrition monitoring methods, most experts enter the farmland to observe leaf color and growth and apply an appropriate amount of nitrogen fertilizer according to the results. However, this method is labor- and time-consuming. To realize automatic rice nitrogen nutrition monitoring, we constructed the Jiangxi rice nitrogen nutrition monitoring model based on a convolution neural network (CNN) using the same region rice canopy image in different generation periods. Our CNN model was evaluated using multiple evaluation criteria (Accuracy, Recall, Precision, and F1 score). The results show that the same CNN model could distinguish the rice nitrogen nutrition status in different periods, which can completely realize the automatic discrimination of nitrogen nutrition status so as to guide the scientific nitrogen application of rice in this area. This will greatly improve the discrimination efficiency of the nitrogen nutrition status and reduce the time and labor cost. The application of the proposed method also proved that the CNN model can be applied in the discrimination of the nitrogen nutrition status. Among CNN models, GoogleNet model proposed a CNN architecture named Inception which can improve the depth of the network and extract higher-level features without changing the amount of calculation of the model. The GoogleNet model achieved the highest accuracy, 95.7%.

## 1. Introduction

Rice is one of the most important food crops in the world. In recent years, owing to the improvement of the breeding level and the scientific use of chemical fertilizers and pesticides, the grain and rice yield has increased year over year [1]. Nitrogen fertilizer is the most important nutrient in the process of fertilization. Too little or too much nitrogen fertilizer will adversely impact the growth of rice [2]. Scientific nitrogen application is beneficial to promote crop growth and strengthen the role of photosynthesis so as to achieve the purpose of increasing yield. To ensure the real-time, rapid, and accurate evaluation of nitrogen fertilizer application in rice, we used the deep learning convolution neural network (CNN) model to realize the automatic classification of the rice canopy image and judge whether the rice in this area is nitrogen-optimal, nitrogen-stressed, or nitrogen-overloaded so as to scientifically apply nitrogen, improve nitrogen use efficiency, prevent nitrogen pollution, and realize the sustainable development of agriculture.

In addition to manual identification, traditional nitrogen content detection also includes leaf color chart technology (LCC) [3], chlorophyll meter technology [4], and remote sensing technology (RS) [5]. Among them, chlorophyll meter technology is only suitable for point detection. Moreover, remote sensing technology is expensive, damages plants, and has a heavy and complex workload. With the improvement of computer vision technology and computer hardware performance, more nitrogen detection methods based on computer vision have been proposed.

Traditional machine learning methods include random forest (RF) [6], K-nearest neighbors (KNN) [7], and support vector machine (SVM) [8]. They mostly manually extract features from existing images and then put the obtained feature values into the machine learning model for training [9]. For example, by manually extracting multiple features (e.g., the color of the rice canopy image), multiple feature classifiers can be used for classification. Owing to lack of texture in multivariable plant images and complicated occlusion and backgrounds, the extracted features cannot fully represent the whole image [10, 11] In addition, this method often relies heavily on fixed datasets, and its generalization ability is poor.

Deep learning CNN technology addresses many of the issues associated with conventional approaches by automatically extracting features from images while combining the feature extraction and classification tasks into a deep learning structure based on CNN. The CNN model has been widely used in agriculture and other fields [12]. There have been a few related studies on the classification of the nitrogen nutrition status using the deep CNN. Azimi S et al. [13] proposed that CNN can learn the features automatically for efficient classification of stress levels in plants using the sorghum plant shoot of a nitrogen-stress public dataset [14]. Owing to the single data set, only 75% accuracy was achieved. An J et al. [15] proposed a CNN model to identify maize drought stress and achieved good results.

At present, there has been no research on nitrogen nutrition monitoring based on the rice canopy image or on the application of deep learning CNN technology to rice nitrogen nutrition monitoring. This study was based on a year of rice canopy image data. The rice canopy image included the tillering stage, jointing stage, and booting stage. Several state-of-the-art CNN models were used for nitrogen nutrition monitoring. The results show that the deep learning CNN model could be completely robust and effectively classify the nitrogen nutrition status of rice in each period. Therefore, this method can completely replace the manual nitrogen nutrition monitoring. It was also superior to other detection methods in practice. Thus, it provides a reference for automatic nitrogen nutritional monitoring of rice.

## 2. Materials and methods

### 2.1 Original image

The experiment was conducted from 2019 to 2020 in Gao’an, Jiangxi Province, China. The experimental station was located at 28°25’27 "N and 115°12’15" E. The rice species provided for the experiment were early rice (*Zhongjiazao 17*), which were treated with three nitrogen levels: 0 kg·ha^−2^, 150 kg·ha^−2^, and 225 kg·ha^−2^. The ratio of base fertilizer to tiller fertilizer to panicle fertilizer was 5:3:2. A visible-light camera *Canon EOS80D* (Canon Inc., Tokyo, Japan) was used to obtain images in the experiment. The image size was 5184*3456 pixels, and the image storage format was jpeg. The camera was set up with the camera lens perpendicular to the rice canopy in the automatic exposure and auto focus mode. At 10:00–14:00 Beijing time, when the solar altitude angle was basically unchanged, the photos were taken at three stages: rice tillering, rice jointing, and rice booting. The rice sampling images of each period are shown in S1 Fig.

Given the large RGB original image, in our experiments, we extracted fixed-size (pixel dimensions of 1024 * 1024) patches by sliding a window of pixel size 1024 × 1024 with a stride of 1024 pixels over an image. We found that only when the patch size was 1024 could we ensure that each patch did not lose nitrogen nutrition status information. Patches with a width or height of less than 1024 were ignored. This left a total number of 15 patches of each original image. For the same nitrogen nutrition status of rice in different periods, patch was classified into a unified category. Our final dataset size was as follows: nitrogen-stressed: 270, nitrogen-optimal: and 270 nitrogen-overloaded: 540. The dataset is divided into a training set and test set according to the ratio of 8:2. We used random geometrical operations (rotations) to balance our training datasets.

### 2.2 Proposed CNN model

In image classification, there are many deep learning CNN algorithms with different structures, each with their own structure and advantages [16, 17]. Their core operation is convolution. Suppose the input image has dimensions (*N*, *C*_in_, *H*_in_, *W*_in_), and the output feature channels have dimensions (*N*, *C*_out_, *H*_out_, *W*_out_), where *N* represents the number of convolution images, the terms involving *C* represent the number of channels of the images, and the terms involving *H* and *W* represent the heights and widths of the images, respectively. Then, the convolution process is conducted as follows:

$$\text{out}\left(N_{i},C_{outj}\right)=\text{bias}\left(C_{outj}\right)+\sum_{k=0}^{C_{in}-1}filter\left(C_{outj},k\right)\Phi input\left(N_{i},k\right),$$ (1)

where Φ represents the convolution operation. Each pixel value *S* (*i*, *j*) of the feature map is given as follows:

$$\text{S}\left(\text{i},\text{j}\right)=\sum_{m}\sum_{n}filter\left(m,n\right)input\left(i+m,j+n\right),$$ (2)

where *m* and *n* represent the width and height of the filter, respectively. Here, *m* = *n* for the present model.

We compared these performances with four currently available state-of-the-art algorithms for our dataset. The overall process of model training and test is shown in S2 Fig. The structural characteristics of each model are as follows:

#### 2.2.1 GoogleNet

Szegedy C et al. [18] proposed a CNN architecture named Inception (S3 Fig). The core idea of the Inception is based on finding out how an optimal local sparse structure in a CNN can be estimated and overlaid by readily available dense components. In this structure, 1 × 1 convolutions are used to compute reductions, which can not only increase the depth of the network and extract higher-level features, but they can also ensure that the amount of calculation of the model remains unchanged. This approach makes full use of computing resources.

#### 2.2.2 DLA net

Yu F et al. [19] proposed a deep layer aggregation (DLA) CNN architecture. DLA has two aggregation structures. One is iterative deep aggregation (S4(a) Fig), which focuses on the step-by-step fusion and refinement of the resolution and scale of aggregation; the other is hierarchical deep aggregation (S4(b) Fig), which focuses on merging the feature structures of various network blocks and channels. The DLA CNN architecture combines iterative deep aggregation and hierarchical deep aggregation to improve the classification accuracy.

#### 2.2.3 ResNet

He K et al. [20] proposed a residual learning CNN architecture to ease the training of networks that are substantially deeper than previous networks. The depth of features is of primary importance for many visual classification tasks. The architecture reformulates the layers as learning residual functions with reference to the layer inputs. This way, the CNN network is easier to optimize, and the accuracy can be obtained from the greatly increased depth. The CNN adopts residual learning in every two or more convolution layers (S5 Fig).

#### 2.2.4 MobileNet

Sandler M et al. [21] proposed an inverted residual architecture where the shortcut connections are between the basic building blocks. The medium expansion layer uses lightweight depthwise convolutions to filter features as a source of non-linearity. The basic building block is bottleneck depthwise separable convolution, which is the key to improving the efficiency of the model. The core idea is to factorize a full convolution operation into two separate layers. With this architecture, the model significantly reduces the calculated amount and memory footprint (S6 Fig).

### 2.3 CNN model training

#### 2.3.1 Device

Proposed CNN models were implemented on a personal computer with the Windows 10 64-bit operating system, an Intel^®^ Core^™^ i7-8700 central processing unit (CPU) operating at 3.20 GHz, and 40 GB of random access memory (RAM), in addition to an NVIDIA GeForce RTX 2060 graphic processing unit (GPU). The development environment was PyCharm. The programming language was Python 3.6.

#### 2.3.2 Data preprocessing

1) The training dataset was randomly cropped to between 8% and 100% of the original region, and the aspect ratio was varied randomly between 3/4 and 4/3, and resized to 224 × 224 pixels by bilinear interpolation [22]. This approach has been demonstrated to improve the robustness of the trained model [23, 24].

2) Images were converted to the type Tensor, and we normalized each channel to a range of 0 to 1. This normalization process was also applied to each color channel of RGB images. These normalization processes were conducted as follows:

$$x=\frac{x-mean\left(x\right)}{std\left(x\right)}.$$ (3)

#### 2.3.3 Training

PyTorch was our CNN framework for model training and testing. Loss function adopted cross-entropy (Eq 4). The optimizer adopted stochastic gradient descent (SGD), with a batch size of 32. The initial lr of training was 0.05 and decreased by a factor of 0.1 every 10 epochs. The model trained 100 epochs in total. After convolution operations, we adopted Batch normalization (Eq 5) to accelerate the convergence of the model [25]. The activation function adopted ReLu (Eq 6) rather than the sigmoid or Tanh function, in conjunction with the SGD method, which has been demonstrated to increase the rate of model convergence [26]. Before fully connecting the network after the last convolution operation, we add an adaptive average pooling layer to the model, which effectively reduces the number of features entering the fully connected layer, model complexity and prediction time.


$$loss(out,label)=-out[label]+\text{log}(\sum_{i=0}^{3}e^{out[i]}).$$ (4)

Here, *out* is the predicted value by the CNN model, and *label* is the actual class of the image.


$$\text{y}=\gamma \frac{x-E\left(x\right)}{\sqrt{Var\left(x\right)}}+\beta .$$ (5)

Here, x represents the standard convolution layer output value, *E*(*x*) represents the mean value of *x*, *Var*(*x*) represents the variance of *x*, and *γ* and *β* are parameters adjusted during the training process.


$$\left\{\begin{array}{c} x\;\;if\;x>0\\ 0\;\;if\;x<0 \end{array}\right..$$ (6)

## 3. Results and discussion

### 3.1 Confusion matrix

To ensure the comparability of the results, the data processing process and training process of four models were consistent. We adopted a confusion matrix to evaluate the performance of the four proposed CNN classification models. As listed in Table 1, if any given class was condition-positive, then all of the other classes were condition-negative. TP represents the number of condition-positive classes predicted correctly by the model. FN represents the number of condition-positive classes predicted incorrectly by the model. FP represents the number of condition-negative classes predicted correctly by the model. TN represents the number of condition-negative classes predicted incorrectly by the model. The results of the four models are shown in S7 Fig.

**Table 1:** Confusion matrix.

		Predicted condition		
	Total population	Prediction positive	Prediction negative	
True condition	Condition positive	True positive (TP)	False negative (FN)	$\text{True}\;\text{positive}\;\text{rate}\left(\text{TPR}\right)=\frac{\sum TP}{\sum \text{Condition}\;\text{positive}}$
	Condition negative	False positive (FP)	True negative (TN)	$\text{False}\;\text{positive}\;\text{rate}(\text{FPR})=\frac{\sum FP}{\sum \text{Condition}\;\text{negative}}$

In addition, we also defined the performance metrics Accuracy, Recall, Precision, and F1 score as in Eqs 7–10. Here, Accuracy reflects the ability of the model to correctly identify the classes from all images. Recall, which is also denoted as the true positive rate (TPR), reflects the ability of the model to correctly identify images corresponding to the positive classes from the entire collection of positive classes. Precision reflects the ability of the model to correctly identify images corresponding to the positive classes from the entire collection of prediction positive classes. Here, Recall and Precision are generally given as the average values obtained for all of the classes. Finally, the F1 score is the weighted average of Precision and Recall. The classification performance of a model increased with increasing values of Accuracy, Recall, Precision, and F1 score.


$$\text{Accuracy}=\;\frac{TP+TN}{TP+FN+FP+TN}\times 100\text{\%},$$ (7)

$$\text{Recall}=\;\frac{TP}{TP+FN}\times 100\text{\%},$$ (8)

$$\text{Precision}=\;\frac{TP}{TP+FP}\times 100\text{\%},$$ (9)

$$\text{F}1\;\text{score}=\;2\times \frac{Precison\times Recall}{Precison+Recall}\times 100\text{\%}.$$ (10)

The results for the four models are listed in Table 2. Avg. Recall or Avg. Precision indicates the metrics calculated for each image, and gives their average value. In addition, the table includes the number of model parameters requiring training, the average time required by a model to classify each image, and the number of multiplication and addition operations required by a model. The highest performances are given in bold font in the table.

**Table 2:** Classification performances obtained with four CNN models for the real-world testing dataset.

Classification method	Accuracy	Avg. Recall	Avg. Precision	F1 score	Parameters (×10^6^)	Time (ms/image)	mult-adds (×10^9^)
GoogleNet	**95.99%**	**95.99%**	**95.98%**	**95.98%**	5.610	19.0	1.51
DLANet	74.54%	74.54%	72.98%	73.28%	15.275	15.4	1.24
ResNet	86.27%	86.27%	86.28%	86.26%	23.528	16.01	4.14
MobileNet	83.64%	83.64%	83.98%	83.78%	**2.236**	**14.26**	**0.1524**

The results of S7 Fig and Table 2 indicate that all models could accurately identify the three statuses of rice nitrogen nutrition, but the GoogleNet model performed better than all the other models in terms of Accuracy, Recall, Precision, and F1 score. Moreover, the MobileNet model was faster than all of the other models because it requires the smallest number of adjustable model parameters and multiplication and addition operations. However, the proposed four models could all process images in real time, which indicates that our model can meet the needs of actual N nutrition detection regardless of speed or accuracy.

### 3.2 Receiver operating characteristic curve and area under the curve

The receiver operating characteristic (ROC) curve and the area under the curve (AUC) are also widely used measures of CNN classification model performance [27]. The value of the abscissa of the ROC curve is FPR and the value of the ordinate is TPR. Table 1 indicates that FPR reflects the ability of the model to correctly identify images corresponding to the negative classes from the entire negative images. The AUC feature has been demonstrated to represent the degree of confidence that can be ascribed to a CNN model for obtaining a correct classification prediction [28]. The larger the AUC value, the higher the robustness and accuracy of the model in identifying this category of images.

Therefore, the ROC curves obtained for the three classes by the proposed four CNN models in S8 Fig. and their corresponding AUC values are given within the figure legends. The micro-average and macro-average ROC curves obtained for the collection of three classes are also presented in each figure. From S8 Fig, we can conclude that the GoogleNet model achieved the best effect, and the average AUC was 0.99.

## 4. Conclusions

The present study addressed the inefficiencies, high cost, and destructiveness of current detection methods by CNN models for conducting nitrogen nutrition classification automatically and accurately based on the rice canopy image, and it showed the proposed method to be an efficient addition to nitrogen nutrition monitoring systems. The proposed model applies CNN technology directly to a canopy image (RGB image). The model automatically extracts the advanced features of the image through CNN, which does not need to extract the features manually, so as to ensure the integrity of the features. The accuracy and efficiency of the proposed CNN models were demonstrated for a real-world dataset. According to the quantitative results obtained, we can conclude that the proposed GoogleNet CNN model offers better classification performance than the other CNN models considered owing to better robustness and generalization ability. Thus, the GoogleNet model can be applied to the real-time nitrogen nutrition monitoring equipment in practical rice planting.

## Supporting information

S1 Fig Early rice RGB images. (a) tillering nitrogen stress (b) tillering nitrogen optimum (c) tillering nitrogen overload (d) jointing nitrogen stress (e) jointing nitrogen optimum (f) jointing nitrogen overload (g) booting nitrogen stress (h) booting nitrogen optimum (i) booting nitrogen overload.
(TIF)

S2 Fig An overview of the proposed workflow. (TIF)

S3 Fig Inception architecture. (TIF)

S4 Fig DLA architecture (a) iterative deep aggregation (b) hierarchical deep aggregation. (TIF)

S5 Fig Residual learning: A building block. (TIF)

S6 Fig MobileNetV2: A basic building block. (TIF)

S7 Fig Confusion matrix representations of the four models. (a) GoogleNet (b)DLANet (c)ResNet (d) MobileNet.
(TIF)

S8 Fig ROC curve and area AUC of the four models. (a) GoogleNet (b) DLANet (c) ResNet (d) MobileNet.
(TIF)
