# Supplementary figures and images for: Rice nitrogen nutrition monitoring classification method based on the convolution neural network model: Direct detection of rice nitrogen nutritional status
Source: PLoS One. 2022 Nov 22;17(11):e0273360. doi: 10.1371/journal.pone.0273360 (PMC9681082; doi:10.1371/journal.pone.0273360)

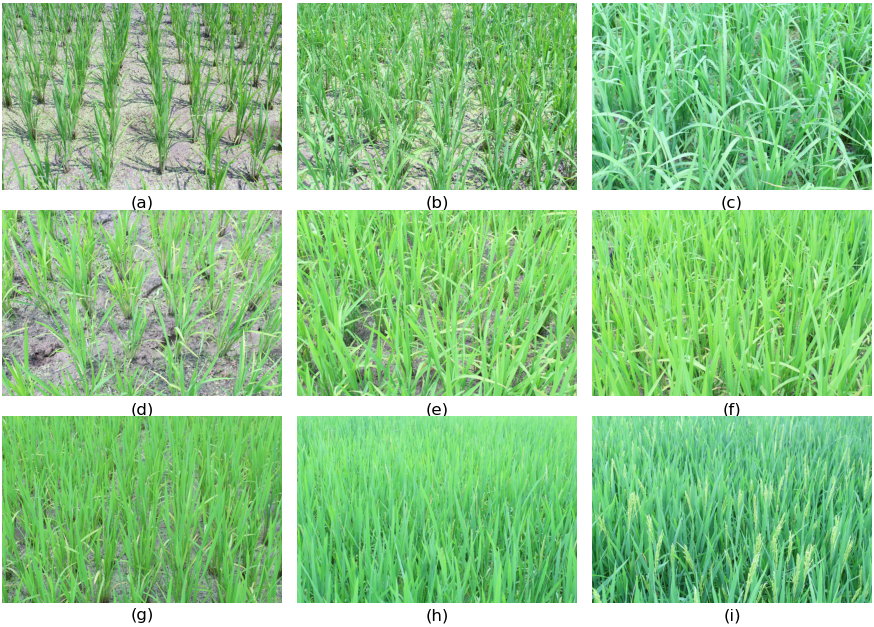

Supplement: S1 Fig — (a) tillering nitrogen stress (b) tillering nitrogen optimum (c) tillering nitrogen overload (d) jointing nitrogen stress (e) jointing nitrogen optimum (f) jointing nitrogen overload (g) booting nitrogen stress (h) booting nitrogen optimum (i) booting nitrogen overload. (TIF) [file pone.0273360.s001.tif]

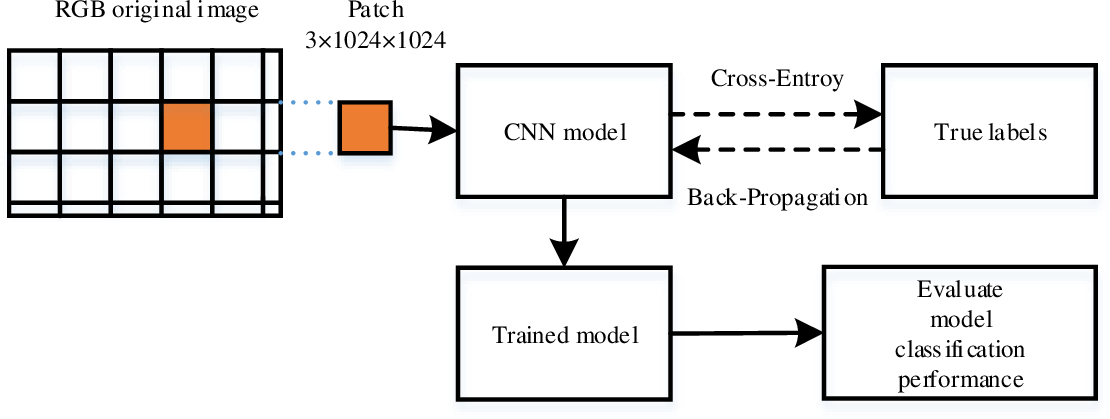

Supplement: S2 Fig — (TIF) [file pone.0273360.s002.tif]

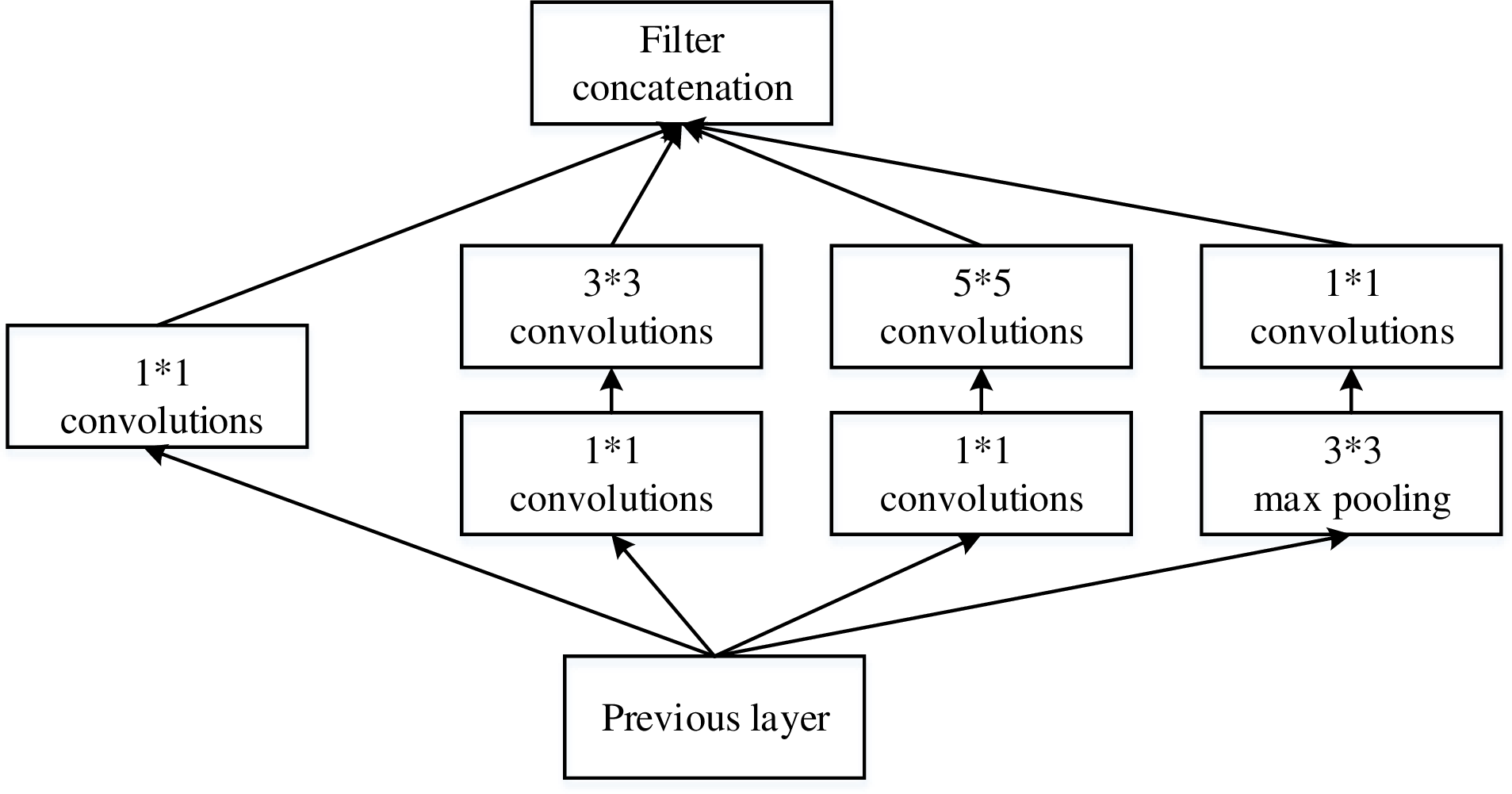

Supplement: S3 Fig — (TIF) [file pone.0273360.s003.tif]

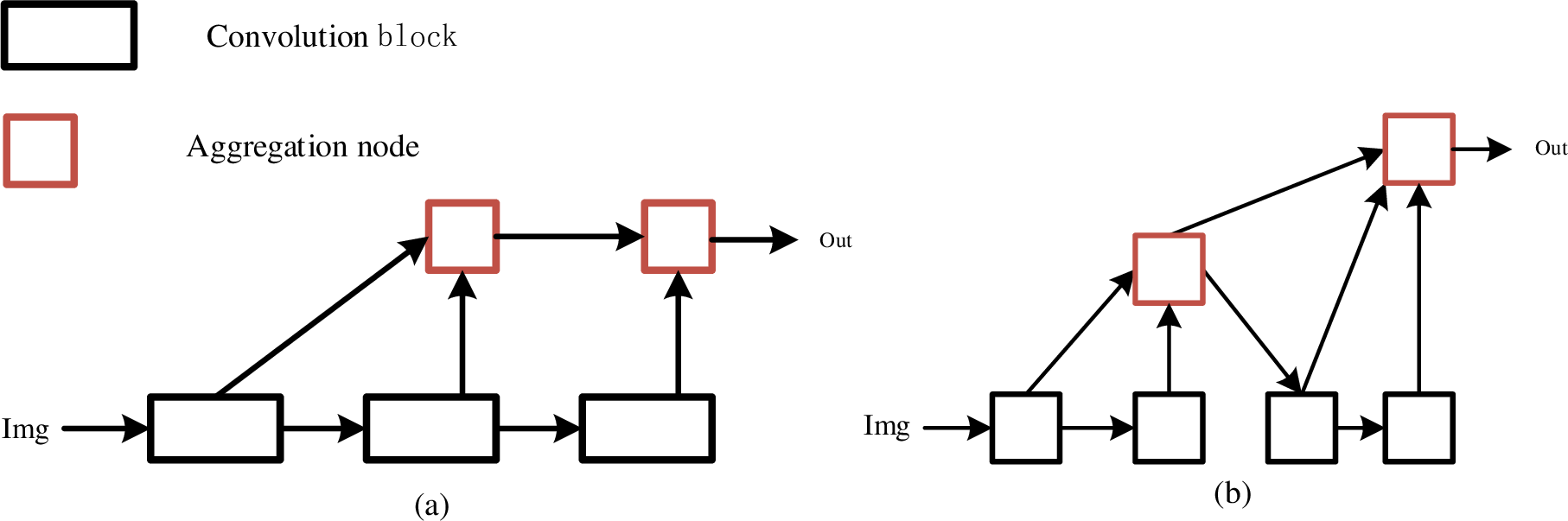

Supplement: S4 Fig — (TIF) [file pone.0273360.s004.tif]

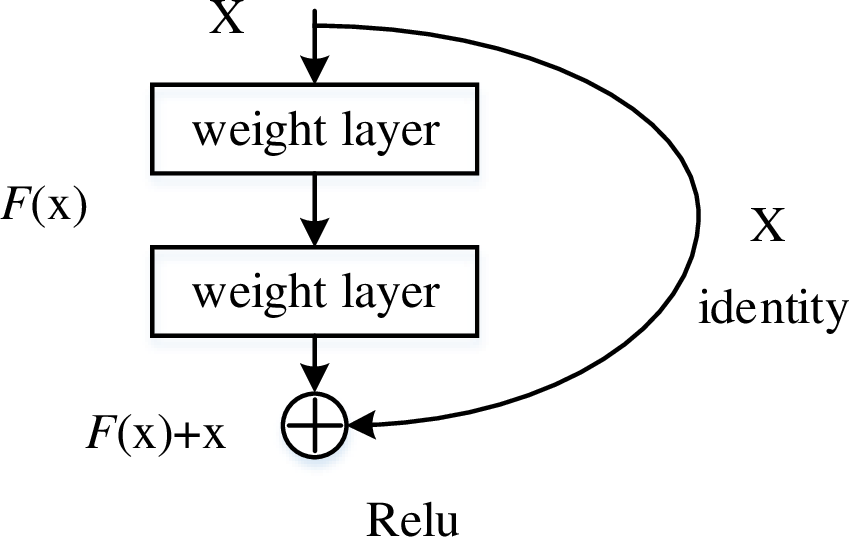

Supplement: S5 Fig — (TIF) [file pone.0273360.s005.tif]

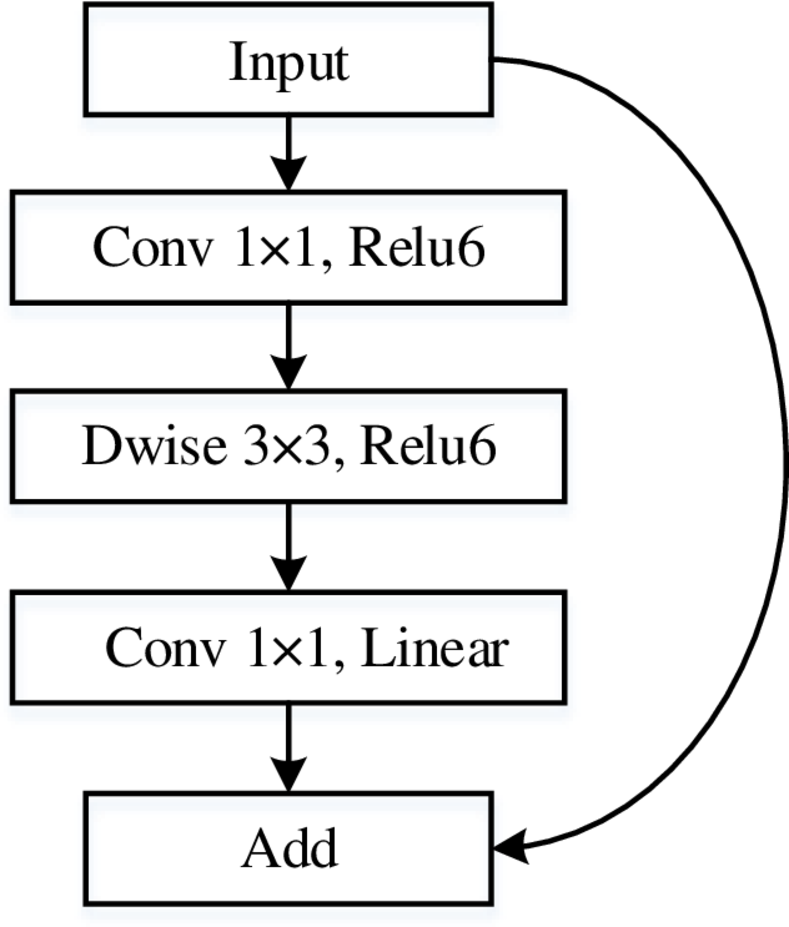

Supplement: S6 Fig — (TIF) [file pone.0273360.s006.tif]

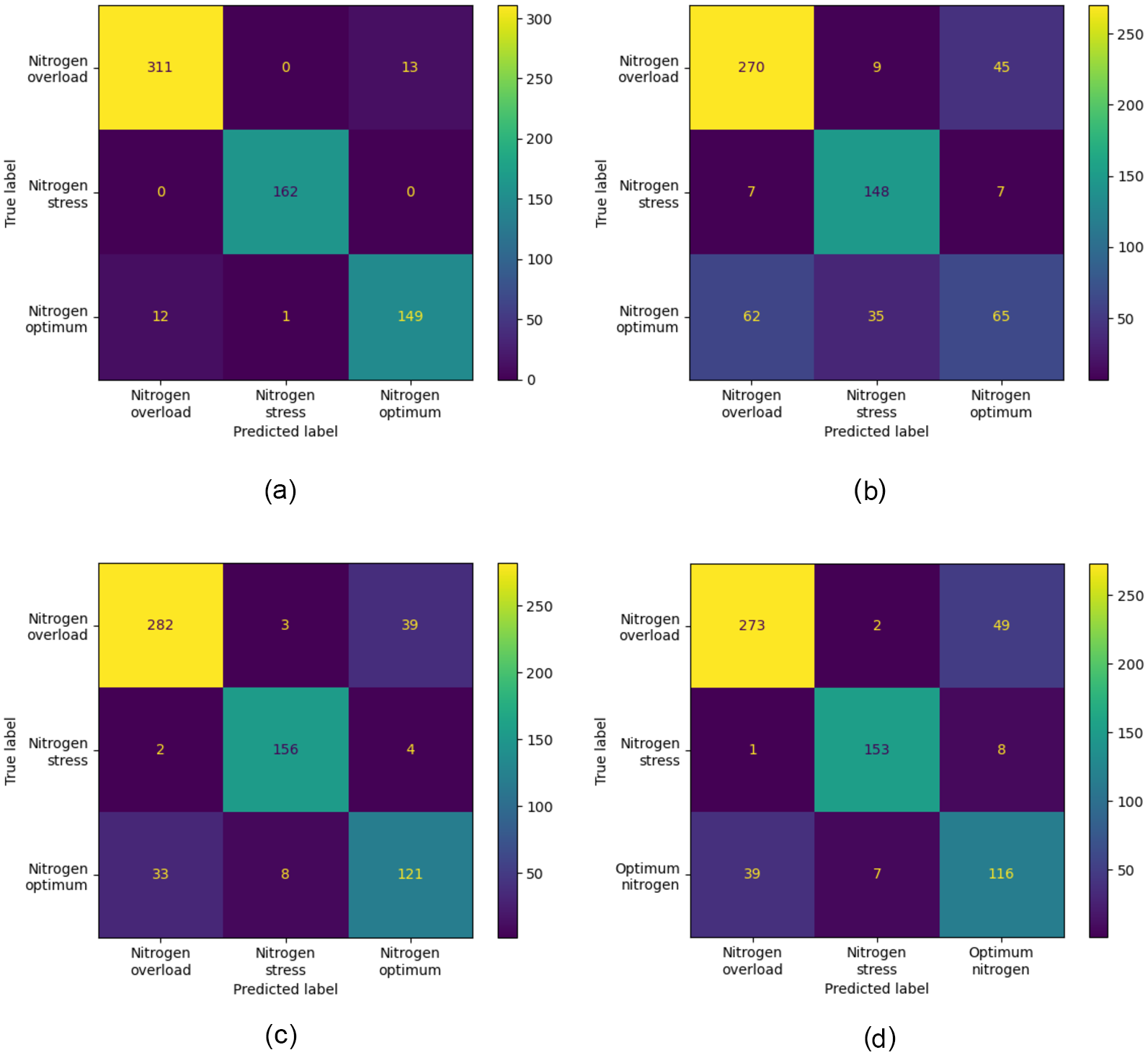

Supplement: S7 Fig — (a) GoogleNet (b)DLANet (c)ResNet (d) MobileNet. (TIF) [file pone.0273360.s007.tif]

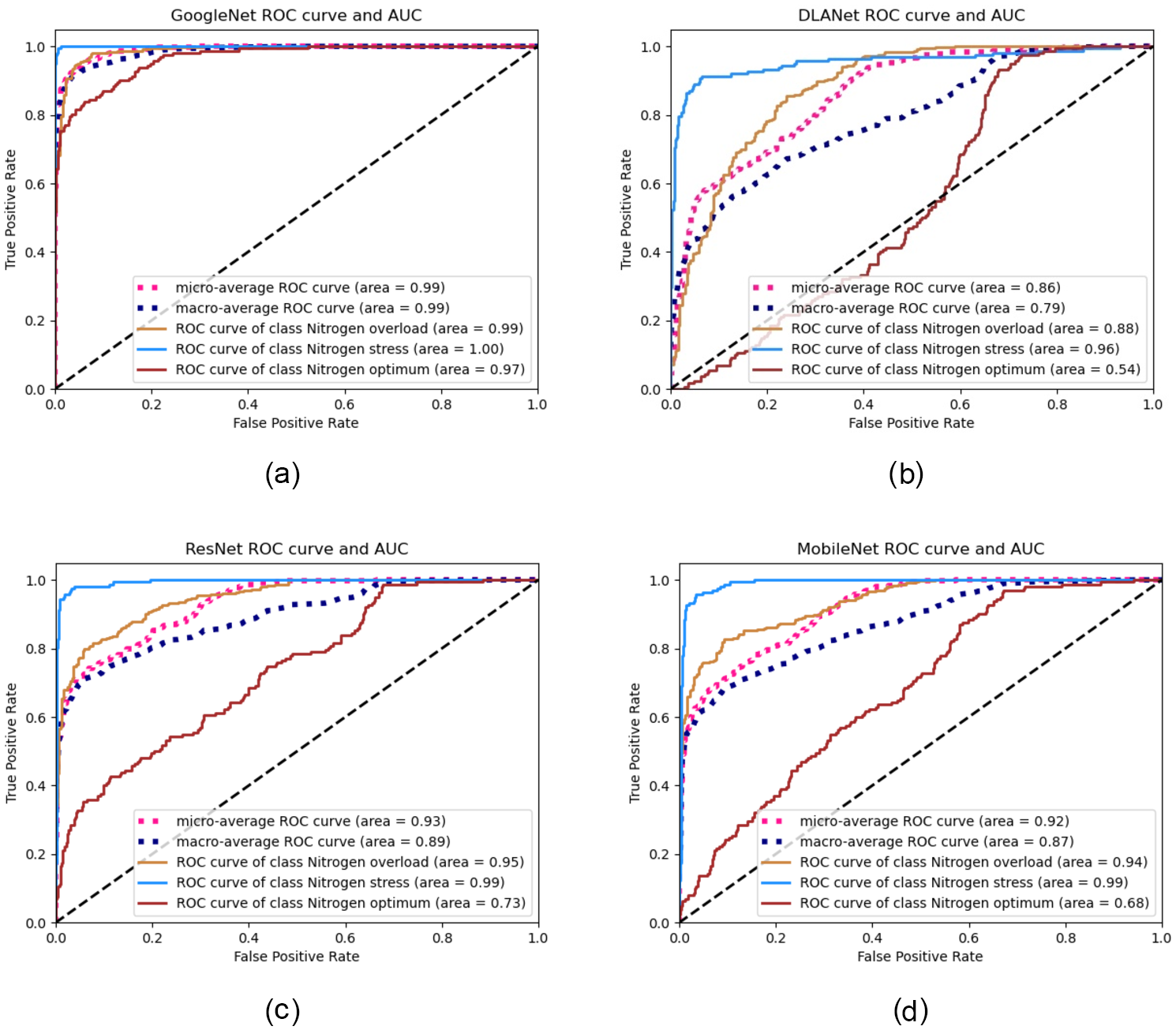

Supplement: S8 Fig — (a) GoogleNet (b) DLANet (c) ResNet (d) MobileNet. (TIF) [file pone.0273360.s008.tif]
